# Response of Pea Plants (*Pisum sativum cv. Ran 1*) to NaCl Treatment in Regard to Membrane Stability and Photosynthetic Activity

**DOI:** 10.3390/plants12020324

**Published:** 2023-01-10

**Authors:** Antoaneta V. Popova, Preslava Borisova, Dimitar Vasilev

**Affiliations:** Institute of Biophysics and Biomedical Engineering, Bulgarian Academy of Sciences, 1113 Sofia, Bulgaria

**Keywords:** salt stress, *Pisum sativum*, membrane stability, photosynthetic performance, primary photosynthetic reactions

## Abstract

Salinity is one of the most extreme abiotic stress factors that negatively affect the development and productivity of plants. The salt-induced injuries depend on the salt tolerance of the plant species, salt concentration, time of exposure and developmental stage. Here, we report on the response of pea plants (*Pisum sativum* L. *cv Ran 1*) to exposure to increasing salt concentrations (100, 150 and 200 mM NaCl) for a short time period (5 days) and the ability of the plants to recover after the removal of salt. The water content, membrane integrity, lipid peroxidation, pigment content and net photosynthetic rate were determined for the pea leaves of the control, treated and recovered plants. Salt-induced alterations in the primary photosynthetic reactions and energy transfer between the main pigment–protein complexes in isolated thylakoid membranes were evaluated. The pea plants were able to recover from the treatment with 100 mM NaCl, while at higher concentrations, concentration-dependent water loss, the disturbance of the membrane integrity, lipid peroxidation and an increase in the pigment content were detected. The net photosynthetic rate, electron transport through the reaction centers of PSII and PSII, activity of PSIIα centers and energy transfer between the pigment–protein complexes were negatively affected and were not restored after the removal of NaCl.

## 1. Introduction

Salinity is one of the most detrimental abiotic stress conditions, and it is engendered by the overuse of fertilizers, unsustainable use of agricultural lands, application of incorrect irrigation practices and climate change [1,2]. A high salt content negatively impacts the development of plants due to the retardation of the plant’s growth, ion balance and water content [3,4,5] and seriously decreases the yields of crops. More than 6% of the world’s total lands and more than 20% of the irrigated agricultural areas in the world contain high amounts of different salts [6,7]. The ever-growing world population and increasing need for food has rendered the necessity of increasing the yields of agricultural products and the development of strategies for improving the salt tolerance of crops highly appropriate. It is of key importance to investigate, in detail, the mechanisms of salt-induced injuries affecting plants that are not completely understood based on the investigation of the responses of vast variety of plants and crops to salt, different concentrations of various salts and short and long terms of exposure.

The extent of salt-induced injury is dependent on a number of factors, such as the type and concentration of a particular salt, the duration of treatment and the salt tolerance of the respective plant species [8,9]. With respect to salt tolerance, plants are arranged into two main groups: halophytes that can develop at increased salt concentrations and glycophytes, including the majority of crop species, that are negatively affected by salts [6,10]. The salt-induced inhibition of plant development and productivity is mainly due to osmotic stress, that is, the initial, fast and reversible phase of salt stress, and ion toxicity, being the second, slow and irreversible phase [11], followed by secondary stress-related processes, mainly due to the excessive generation of ROS [12]. The salinity of soil significantly reduces the ability of plants to absorb water, thus leading to osmotic stress expressed as membrane interruption, nutrient imbalance, the disturbance of enzyme antioxidant and photosynthetic activities and decreases in stomatal aperture. The extent of plants’ sensitivity to increased salt concentrations is higher in their early stages of development, such as germination and the seedling stage [6,8,11,13,14]. The second and more detrimental phase of salt stress is the accumulation of Na^+^ and Cl^-^ ions in plant cells reaching toxic levels that can lead to serious physiological disbalances, the intensive production of reactive oxygen species (ROS), premature senescence and plant death [6,14,15,16,17].

The photosynthetic machinery is one of the components of photosynthetic organisms most sensitive to detrimental environmental conditions, including salinity. However, reports on the impact of salt stress on the functioning of the photosynthetic process are still rather limited [11,18].

The photosynthetic pigment content is seriously affected by salt imposition. A decline in chlorophyll has been reported in plants such as sunflower [19], alfalfa [20], wheat [21], tomato [22], etc. However, there are also reports stating that the chlorophyll content can be increased after exposure to salt treatment, mainly in salt-tolerant taxa [23,24,25]. Carotenoids are also differentially affected by salt stress. A decline in the chlorophyll and carotenoid contents was registered in sugar cane, while in salt-tolerant varieties, increased levels of pigments were detected [26,27].

Under light conditions, photosystem I (PSI) and photosystem II (PSII) are the main sources of ROS generation [28,29], which is significantly accelerated under salinity conditions in both sensitive and tolerant species [30]. Salt stress negatively affects the functioning of PSII by inhibiting the transfer of electrons on its donor side (oxygen-evolving complex—OEC) [3] between Q_A_ and Q_B_ on the acceptor side [11], as well as the energy transfer between the light harvesting complex of PSII (LHCII) and the reaction center of PSII [31]. The activity of PSI was not found to be significantly affected by NaCl, while other sodium salts resulted in an inhibitory effect [32].

The most susceptible to stress conditions is the multiprotein complex of PSII and its donor side, OEC [33]. It was considered that there are two populations of PSII that are different in terms of their structure and functionality: PSIIα and PSIIβ [34,35,36]. The PSIIα centers are located in the grana domains of thylakoid membranes, possess larger antenna, form dimmers and contain a functional acceptor and donor side [34], while the PSIIβ centers are situated in the stroma-exposed thylakoid lamellae, possess smaller antenna and are organized as monomers. Both types of PSII centers demonstrate different levels of sensitivity towards stress stimuli, including heat and altered fluidity [37], low temperatures [38] and UV treatment [39].

Pea (*Pisum sativum* L.) is one of the most widely planted crops that is seriously affected by NaCl concentrations higher than 100 mM [40,41]. Pea species vary with respect to salt tolerance and are classified as less, intermediate and highly salt tolerant [42]. In NaCl-sensitive cv. (Challis), increased levels of H_2_O_2_ and lipid peroxidation were detected, the chlorophyll content and PSII activity were significantly decreased, and the chloroplast integrity was disorganized. However, the tolerant taxa (cv. Granada) responded to salt stress with increased levels of antioxidant enzyme activity, increased ascorbate content and increase in the number and size of the plastoglobuli [24]. The salt-induced alterations in the water relations and activity of antioxidant enzymes after short- (48 h) [43] and long-term (15 days) [44] exposure to 70 mM NaCl indicated the salt-induced elevated generation of ROS. Salt-induced physiological rearrangements in pea plants after two weeks of exposure to 80 mM NaCl facilitated the survival of the plants under these conditions [45].

The aim of the present study was to follow the response of *Pisum sativum* L. *cv. Ran 1* to salt stress imposed by increased concentrations NaCl (100, 150 and 200 mM) for a short period (5 days) and the ability of plants to recover after the removal of salt from growth medium. The water status of the plants, integrity of biological membranes, extent of lipid peroxidation and the pigment content were estimated using pea leaves. The impact of salt stress on the photosynthetic oxygen evolution in vivo was evaluated using light-response curves of leaf discs. Alterations in the primary photosynthetic reactions were determined in isolated thylakoid membranes from control, treated and recovered pea plants.

## 2. Results

### 2.1. Salt-Induced Alterations in Water Status, Electrolyte Leakage and Lipid Peroxidation of Pea Plants

**Figure 1:**
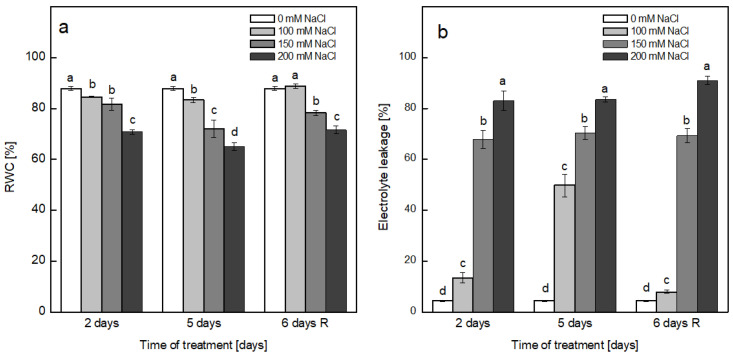
Salt-induced alterations in the relative water content (RWC) (**a**) and electrolyte leakage (EL) (**b**) of the leaves of pea plants after treatment with 0, 100, 150 and 200 mM NaCl for 2 and 5 days and after recovery (6 days R) on pure Hoagland’s solution. Results are presented as means ± SE. Three independent experiments were performed with 3 parallel samples at each time point (*n* = 9). Different letters indicate significant differences between the values for the control plants (0 mM NaCl) and plants treated with increasing concentrations of NaCl at *p* < 0.05 for the respective period of the experimental setup (2 and 5 days of treatment and the recovery period).

The negative effect of the salt treatment with increased concentrations of NaCl on pea plants over two time periods (2 and 5 days) and their ability to restore after the stress were evaluated in leaves from the control, treated and recovered plants. The relative water content (RWC) in the leaves of pea plants was gradually decreased through the elevation of the salt concentration by 4, 7 and 20% on the second day of treatment, with 100, 150 and 200 mM NaCl, respectively. For the longer period of treatment, the decline in RWC was more strongly expressed, attaining 74.2% for the highest concentration of salt in comparison with the control plants. Omitting NaCl and further development of the plants on pure Hoagland’s solution, a complete recovery of RWC was observed only for the plants that were previously treated with 100 mM NaCl. For the higher concentrations of NaCl, an improvement of several percent in RWC was detected in comparison with the plants assessed after treatment for 5 days (Figure 1a).

The integrity of cellular membranes, which was affected by salt stress, was evaluated by the extent of electrolyte leakage (EL) from the leaves of the pea plants (Figure 1b). The leaves of non-treated plants showed very low levels of EL (4.3%). A time-dependent destabilization of the membranes was detected in the presence of 100 mM NaCl, expressed as elevations to 13.4 and 47.7% after 2 and 5 days of treatment, respectively. The removal of NaCl led to a significant decrease in EL reaching 8%, indicating the near-complete recovery of the membrane integrity after treatment with the indicated concentration. Exposure to higher concentrations of NaCl (150 and 200 mM) caused an intensive leakage of the cell contents that was comparable between the two periods of treatment and the recovery period. The percentages of EL after treatment with 150 mM NaCl were 67.8, 70.3 and 69.2% for the second and fifth day of treatment and for the recovered plants, respectively. The destabilization of membranes was highest after treatment with 200 mM NaCl, reaching 83.0, 83.4 and 90.9% on the 2nd and 5th days of treatment and after the recovery period, respectively, suggesting that no recovery of the membrane integrity was detected after treatment with these NaCl concentrations.

**Figure 2:**
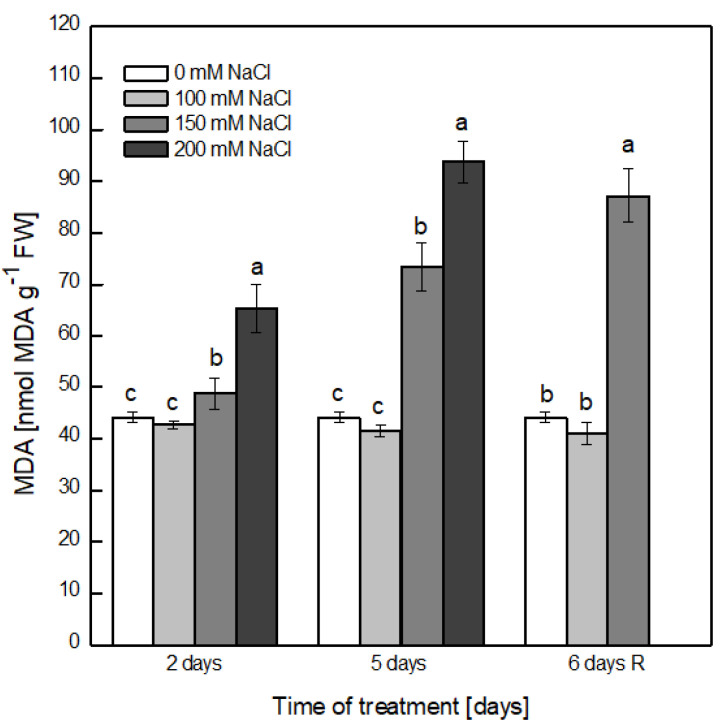
Extent of lipid peroxidation determined by the level of malondialdehyde (MDA) formation in the leaves of pea plants treated with 0, 100, 150 and 200 mM NaCl for 2 and 5 days and after recovery (6 days R). Results are presented as means ± SE. In three independent experiments, four parallel samples were evaluated at each time point (*n* = 12). Different letters indicate significant differences between values for the control plants (0 mM NaCl) and plants treated with increased concentrations of NaCl at *p* < 0.05 for the respective period of the experimental setup (2 and 5 days of treatment and the recovery period).

Salt-induced lipid peroxidation was evaluated by the level of malondialdehyde (MDA) formation (Figure 2). The treatment of the plants with 100 mM NaCl did not lead to an increase in the MDA content during the whole experiment, suggesting that no lipid peroxidation was induced at this concentration. Higher NaCl concentrations (150 and 200 mM NaCl) progressively elevated the MDA content by 10% and 48%, respectively, on the second day of treatment in comparison with control plants of the same age. Longer exposure to salt stress elevated the extent of lipid peroxidation to 66% and 85% at 150 and 200 mM NaCl, respectively. After the recovery period, the plants treated with 150 mM NaCl still demonstrated a very high level of MDA (87%) that was higher than the level after 5 days of treatment with the same concentration of NaCl, indicating some residual stress-related processes.

### 2.2. Pigment Content in the Leaves of Pea Plants after Salt Treatment

**Figure 3:**
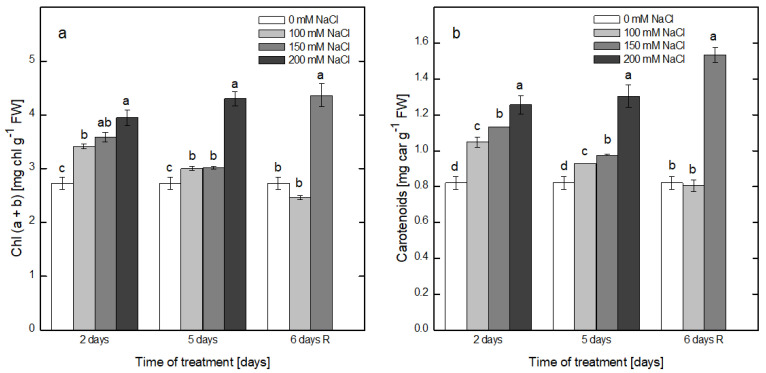
Effect of treatment with increased concentrations of NaCl (0, 100, 150 and 200 mM) on the leaf pigment content of the pea plants treated with the respective concentration for 2 and 5 days and after recovery on pure Hoagland’s solution (6 days R). Results are presented as means ± SE. Three independent experiments were performed, and four parallel samples were evaluated at each time point (*n* = 12): (**a**)–chlorophyl a + b (Chl a + b), (**b**)–total content of carotenoids (Carotenoids). Different letters indicate significant differences between values for the control plants (0 mM NaCl) and plants treated with increasing concentrations of NaCl at *p* < 0.05 for the respective period of the experimental setup (2 and 5 days of treatment and the recovery period).

Alterations in the pigment content in leaves of pea plants exposed to treatment with NaCl and after recovery are presented in Figure 3. After 2 days of exposure to NaCl, the content of chlorophyll a + b (Chl a + b) increased gradually from 2.72 ± 0.01 mg Chl g^−1^ FW (fresh weight—FW) in the non-treated plants to 3.41 ± 0.05, 3.59 ± 0.09 and 3.94 ± 0.15 mg Chl g^−1^ FW with the elevation of salt concentration, including 100 mM, 150 mM and 200 mM, respectively. An increase of 45% was detected at the highest concentration of NaCl in comparison with the control leaves. The increases in the Chl a + b content after exposure to 100 and 150 mM NaCl for 5 days were comparable, but in the presence of 200 mM NaCl, the increase was 58% in comparison with that of the control plants. The recovery of the plants after treatment with 100 mM NaCl led to a restoration of the Chl a + b content to values comparable with those of the control. However, the plants that were previously treated with 200 mM NaCl still showed high Chl a + b contents, which were 60% higher in comparison with the non-treated plants (Figure 3a). The same tendency toward salt-induced increase was detected for the carotenoid content in the case of every time point of the experimental setup and every particular salt concentration (Figure 3b).

### 2.3. Oxygen Evolution of the Whole Leaves after the Treatment of Pea Plants with NaCl

**Figure 4:**
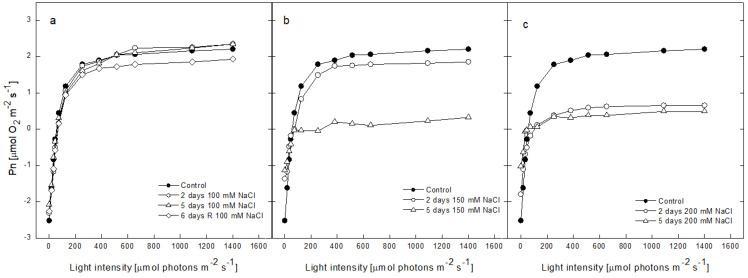
Light response curves of the photosynthetic oxygen evolution of 8 leaf discs of the pea plants, including the control (0 mM NaCl) and those treated with 100, 150 and 200 mM NaCl for 2 and 5 days and after recovery (6 days R) on pure Hoagland’s solution. Measurements were performed at room temperature (22 °C) and in a CO_2_-saturated atmosphere, with increasing light intensities (0–1400 µmol photons m^−2^ s^−1^) provided by an LED source (>650 nm). Every curve is the average from 3 independent experiments with 2 parallel samples (*n* = 6): (**a**)–control plants (Control), treated with 100 mM NaCl for 2 and 5 days and after recovery (6 days R), (**b**)–control plants (Control), treated with 150 mM NaCl for 2 and 5 days and after recovery (6 days R), and (**c**)–control plants (Control), treated with 200 mM NaCl for 2 and 5 days.

The salt-induced alterations in the ability of plants to undergo an effective oxygen evolution were evaluated by constructing light response curves (Figure 4). At low light intensities, the rate of oxygen evolution increased linearly before reaching a plateau at 400 µmol photons m^−2^ s^−1^. No significant differences were observed in the amount of evolved oxygen at all the light levels after the exposure of the pea plants to 100 mM NaCl for 2 and 5 days. After recovery, the level of oxygen evolution was slightly below the curves of the non-treated and treated plants (Figure 4a). The light response curve after treatment with 150 mM NaCl for 2 days was 15% lower than that of the non-treated plants, while after 5 days of treatment, the saturated level of oxygen evolution was 90% lower in comparison with the control (Figure 4b). Treatment with 200 mM NaCl for 2 and 5 days resulted in a similarly low level of oxygen evolution. The plateaus of the light response curves were 70 to 90% lower when compared with the control plants (Figure 4c).

### 2.4. Photochemical Activity of PSII, PSI and Oxygen Evolving Complex (OEC) Affected by the NaCl Treatment

**Figure 5:**
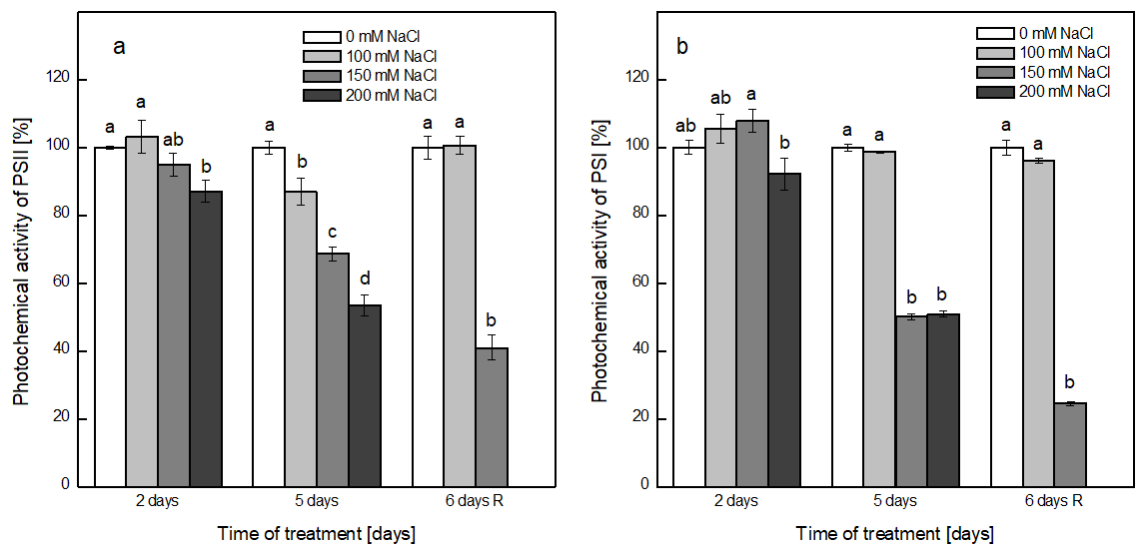
Salt-induced changes in the photochemical activity of PSII (**a**) and PSI (**b**) in isolated thylakoid membranes from the non-treated plants, plants treated for 2 and 5 days with 0, 100, 150 and 200 mM NaCl and recovered pea plants after 6 days (6 days R). Results are presented as the percent of the respective control (0 mM NaCl). Measurements were performed in the presence of exogenous electron donors and acceptors, as described in the Materials and Methods. Results are presented as mean values ± SE, calculated based on three independent experiments with three parallel samples for every time point (*n* = 9): 100% for PSII—36.93 ± 0.18 μmol O_2_ mg Chl^−1^ h^−1^ and for PSI—267.55 ± 5.54 μmol O_2_ mg Chl^−1^ h^−1^. Different letters indicate significant differences between values for the control plants (0 mM NaCl) and plants treated with increased concentrations of NaCl at *p* < 0.05 for the respective period of the experimental setup (2 and 5 days of treatment and after recovery (6 days R)).

Alterations in the photochemical activities of both photosystems after treatment with NaCl were followed by a Clark-type oxygen electrode in the presence of exogenic electron donors and acceptors that enables us to evaluate the effectiveness of the electron flow through the reaction center (Figure 5). The activity of PSII, determined in the presence of benzoquinone (BQ), was gradually inhibited by the presence of NaCl in a concentration-dependent manner for both periods of treatment. A decrease of 13% was observed in the presence of 100 mM NaCl on the fifth day of treatment followed by a complete recovery after transfer to a medium without salt. In the presence of 200 mM NaCl, the detected decline was 13% and 50% for the second and fifth day of treatment, respectively. On removal of salt, the activity of PSII was further decreased in the thylakoid membranes from the plants treated with 150 mM NaCl, decreasing by 57% in comparison with the membranes from the control plants (Figure 5a). The activity of PSI was slightly affected by the presence of NaCl on the second day of treatment, decreasing by only 8% after exposure to 200 mM NaCl (Figure 5b), but after 5 days of growth in the presence of 150 and 200 mM NaCl, a 50% reduction in the activity was detected. Similar to the activity of PSII, the transfer of electrons through PSI was further inhibited after the removal of NaCl in the membranes of plants previously treated with 150 mM NaCl.

**Figure 6:**
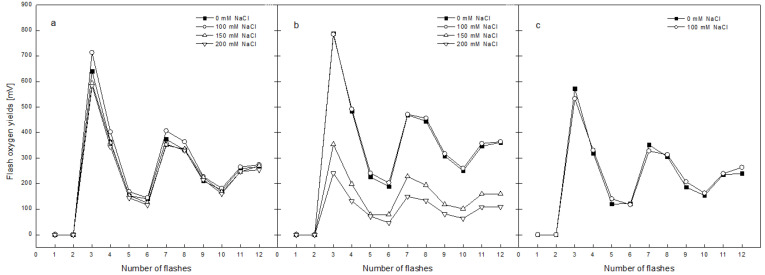
Effect of treatment with different concentrations NaCl (0, 100, 150 and 200 mM) on the flash oxygen yields of pea thylakoid membranes: (**a**)–after 2 days of treatment, (**b**)–after 5 days of treatment and (**c**)–after recovery for 6 days on pure Hoagland’s solution. Mean values ± SE were calculated based on three independent experiments with two parallel samples for every time point (*n* = 6). The flash oxygen yields were calculated using software based on the fitting of the experimentally obtained values to the theoretically calculated oxygen burst yields according to the non-cooperative Kok’s model.

The flash-induced oxygen yields and oxygen burst under continuous illumination without the addition of electron acceptors were recorded for the evaluation of salt-induced alterations in the OEC activity. Figure 6 includes the flash-induced oxygen yields related to the activity of the PSIIα centers located in the grana regions of the thylakoid membranes in isolated thylakoids from non-treated plants (0 mM NaCl) and plants treated with different concentrations of NaCl for 2 days (Figure 6a), 5 days (Figure 6b) and after the recovery period (Figure 6c) of pea plants. All the investigated samples showed a typical oscillation pattern of flash oxygen yields, with the maximum levels at the third and seventh flash. For the second day of treatment with NaCl, the pattern of oxygen flash yields was very similar in terms of intensity. Only a slight decrease of around 7% was detected after the plants’ exposure to 150 and 200 mM NaCl (Figure 6a). Treatment with 100 mM NaCl for 5 days resulted in an identical pattern of oxygen flash yields to that of the thylakoid membranes of non-treated plants of the same age. The growth of the plants over the same period in the presence of 150 and 200 mM NaCl led to a progressive decline in the flash yields by 55% and 70% in comparison with the membranes of non-treated plants, respectively (Figure 6b). The thylakoids from plants recovered from the treatment with 100 mM NaCl showed the same pattern and intensity as those of thylakoids of the control plants (Figure 6c), indicating a complete recovery.

**Figure 7:**
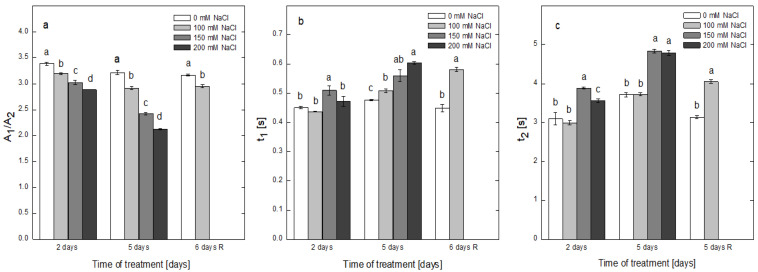
Effect of salt stress on the ratio between the amplitudes of the ‘‘fast’’ (A_1_) and ‘‘slow’’ (A_2_) components of the decay kinetics of initial oxygen burst under continuous illumination related to the PSIIα and PSIIβ centers (**a**) and respective time constants of (t_1_) (**b**) and (t_2_) (**c**), calculated after fitting the decay kinetics with two exponential components in the thylakoid membranes from the non-treated plants (0 mM NaCl) and plants treated with different concentrations of NaCl for 2 and 5 days and after recovery (6 days R). Calculations were performed through the analysis of second-order exponential decay kinetics. Mean values ± SE were calculated based on three independent experiments with two parallel samples for every time point (*n* = 6). Different letters indicate significant differences between values for the control plants (0 mM NaCl) and plants treated with increased concentrations of NaCl at *p* < 0.05 for the respective period of the experimental setup (2 and 5 days of treatment and the recovery period).

The effects of salt stress on the contributions of the two populations of PSII, the “fast” (PSIIα) and “slow” (PSIIβ) centers, to the overall oxygen evolution were evaluated by recording the induction of oxygen burst under continuous illumination without the addition of artificial electron acceptors. The initial oxygen burst was followed by a second-order decay kinetics that could be fitted by a function with two exponential components, providing the amplitudes A_1_ and A_2_ and time constants t_1_ and t_2_, characterizing the functioning of the “fast” and “slow” PSII reaction centers, respectively. The A_1_/A_2_ ratio (Figure 7a) of the thylakoids of plants treated with different concentrations of NaCl showed a gradual, concentration-dependent decrease that proceeded faster for the longer period of exposure to NaCl, suggesting a decrease in the contribution of PSIIα centers to the overall oxygen evolution. For the second day of treatment, the decline was 6, 10 and 15% in comparison with the control plants exposed to 100, 150 and 200 mM NaCl, respectively, while for the longer period of treatment the decline was stronger expressed, decreasing by 10, 25 and 34%, respectively.

Alterations in the kinetic properties of both populations of PSII induced by the salt treatment were evaluated by comparing the two time constants of PSIIα and PSIIβ (t_1_ and t_2_) (Figure 7b,c). The values of t_1_ were elevated with the increase in the salt concentration and time of treatment, reaching 0.61 s after 5 days of treatment with 200 mM NaCl. After recovery, the t_1_ of the plants treated with 100 mM NaCl further increased, reaching a value of 0.582 s, being much higher in comparison with that of thylakoids from control plants (Figure 7b). As expected, the values of the time constant t_2_ of the “slow” PSII centers in thylakoids of the control plants were much higher than t_1_ values of 3.098, 3.712 and 3.139 s for the plants on the second and fifth days of treatment and after recovery, respectively. With the increase in NaCl concentration and time of treatment, the t_2_ constant followed an increasing trend similar to the “fast” constant t_1_ (Figure 7c), indicating that the turnover of both populations of PSII centers was slowed down by the salt treatment.

### 2.5. Salt-Induced Energy Distribution between the Main Pigment–Protein Complexes (77K Fluorescence)

**Figure 8:**
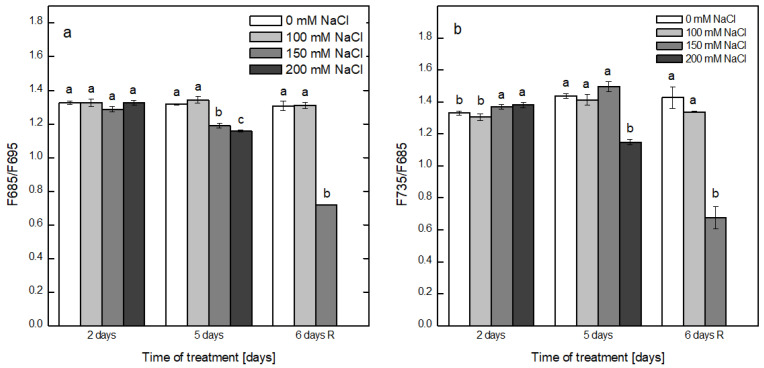
Alterations in the fluorescence F685/F695 (**a**) and F735/F685 ratios (**b**) in the thylakoid membranes isolated from the non-treated plants (0 mM NaCl) and pea plants treated with NaCl (100, 150 or 200 mM) for 2 and 5 days and after recovery (6 days R). Ratios were calculated from the fluorescence emission spectra subjected to excitation with 436 nm. Mean values ± SE were calculated based on three independent experiments with two parallel samples for each time point (*n* = 6). Different letters indicate significant differences between values for the control plants (0 mM NaCl) and plants treated with increased concentrations of NaCl at *p* < 0.05 for the respective period of the experimental setup (2 and 5 days of treatment and the recovery period).

The energy delivery to PSII and PSI in the thylakoid membranes from pea plants was assessed by recording and analyzing the low temperature (77K) fluorescence emission spectra after excitation with 436 nm (the preferential excitation of Chl a). In the emission fluorescence spectra of the thylakoids at 77K, two main fluorescence emission peaks were resolved: one at 685 nm, emitted by the reaction center of PSII, and another at 735 nm, emitted by the complex of PSI and a shoulder at 695 nm emitted by CP47 (the inner antenna of PSII). The energy transfer within the complex of PSII was evaluated by the fluorescence ratio F685/F695 while the transmission of the absorbed energy from the LHCII-PSII to PSI complex was estimated by the ratio F735/F685 (Figure 8a,b). The exposure of plants to NaCl for 2 days did not influence the energy transfer in the PSII complex (Figure 8a). The increased time of treatment led to a gradual decline in the ratio in thylakoids from plants maintained at 150 and 200 mM NaCl. After the recovery period, F685/F695 further decreased in the thylakoids from plants treated with 150 mM NaCl, reaching 0.72 (Figure 8a). The F735/F685 ratio of the thylakoids from non-treated plants showed values of 1.33 ± 0.01 (second day) and 1.43 ± 0.01 for 5th day of treatment and after recovery. After the treatment of plants with 100 mM NaCl for both periods, no significant alteration in F735/F685 was detected, while the application of 150 mM NaCl resulted in a slight increase after 5 days of treatment in comparison with that of control samples. After 5 days, the application of 200 mM NaCl resulted in a decrease in F735/F685 to 1.14 ± 0.02. Omitting NaCl from the growth medium resulted in a significant decrease in the F735/F685 of plants that were treated with 150 mM NaCl to 0.68 ± 0.68 (Figure 8b).

## 3. Discussion

The development of plants and crops in soils with a high content of soluble salts seriously affects their development and productivity on multiple levels, ranging from their growth and morphology to metabolic and physiological processes, including their photosynthetic performance, enzymatic activity and gene expression [11,14,18,46]. Salinity stress occurs in two main phases, including osmotic stress, ion toxicity, followed by secondary stress in plants [11,12]. The osmotic stress is the initial, rapid phase of salt stress sensing. The uptake of water by the roots is seriously reduced, and plants sense a water deficit and loss [4,11,14]. The second, slow phase is related to the accumulation of ions (Na^+^ and Cl^−^) to toxic levels in the cells, leading to ionic disbalances, the disturbance of ion homeostasis, premature senescence and the death of plants [6,14]. The loss of water and accumulation of salt ions in the plant cells are followed by secondary stress processes and the generation of ROS and oxidative stress [12,47].

### 3.1. Alterations in the RWC, Membrane Stability and Lipid Peroxidation

The exposure of pea plants to increased concentrations of NaCl negatively affected the water status of the leaves in a concentration-dependent manner, as demonstrated by a decrease in RWC that was more strongly expressed after treatment for 5 days. The RWC was least affected by treatment with 100 mM NaCl, and the plants were able to restore their water content after the recovery period, while at higher concentrations, the plants were not able to regain their leaf turgor with respect to the level of the control plants.

Biological membranes are the primary target of damage under environmental stress conditions, including salt stress, demonstrated by the disturbance of the membrane integrity and peroxidation of the membrane lipids as result of oxidative stress [24,48]. The extent of electrolyte leakage from the leaf tissues from plants exposed to 100 mM NaCl was elevated with the increase in time of exposure, reaching 50% after 5 days, but the removal of NaCl from the medium resulted in a near-complete restoration of membrane integrity, indicating that treatment with 100 mM NaCl for the indicated period was not detrimental. At the same concentration of NaCl, no increase in lipid peroxidation was detected for the whole experimental period. Similar results regarding the reversible effects of 70 mM NaCl for a short period of treatment (48 h) and 48 h post-stress were reported in pea plants with respect to their growth, leaf water relations and some enzymatic activities, and it was suggested that in the first few hours of both the application and removal of salt, the generation of ROS took place, leading to oxidative stress [43]. Furthermore, it was suggested that the overproduction of H_2_O_2_ in the chloroplasts of salt-sensitive taxa of pea plants treated with 70 mM NaCl for 14 days may be involved in salt damage [24]. No change in the leaf water potential or proline and MDA contents were detected in *Portulaca oleracea* L. after 3 days of exposure to 100 mM NaCl [49]. The higher concentrations of NaCl induced a significant leakage of cell contents, and no considerable improvement was detected after the recovery, indicating that 150 and 200 mM NaCl severely damaged the membrane integrity. The presence of 150 mM NaCl strongly increased the level of lipid peroxidation after 5 days, and this was even further elevated after the recovery period. In the presence of 200 mM NaCl, after 5 days, the extent of lipid peroxidation was significantly increased in comparison with non-treated plants.

These results indicated that the imposition of 100 mM NaCl resulted in a small effect on the turgor of pea leaves and significant disturbance of the membrane integrity, but the observed negative effects were reversible upon removal of salt from the growth medium. The indicated concentration of NaCl did not induce the elevation of lipid peroxidation for the investigated period of treatment. The higher concentrations of NaCl led to a significant extent of water loss, destabilization of the membranes and intensive lipid peroxidation that were not restored after the removal of salt. It can be assumed that the ROS formed in response to 100 mM NaCl were successfully scavenged by the antioxidant system of the plants, including both the non-enzymatic and enzymatic, while in the presence of higher concentrations of NaCl, the antioxidant protection was not effective and/or the accumulated ions in plant cells reached a toxic level and induced ion toxicity.

### 3.2. Pigment Content and Oxygen-Evolving Ability of the Leaves of Pea Plants

Salt stress decreased the pigment content, as reported for sunflower [19,50], alfalfa [20], wheat [21], tomato [22], etc., and its extent was dependent on the tolerance of the respective species or genotype and on the time of the treatment. However, in salt-tolerant species, an elevation in the pigment content was detected [26,50]. Our results indicated that pea plants responded to exposure at increasing concentrations of NaCl for a short period of time with increased pigment contents, which followed a similar trend for both the chlorophylls and carotenoids. This result could be due to either the short period of treatment (5 days), in comparison with the reported times of treatment of tomato (86 days) [22] and pea (15 days) [24], and/or the altered leaf morphology in response to the NaCl treatment, resulting in smaller, thicker leaves and a higher chloroplast density per unit area [6].

After treatment with 100 mM NaCl, no significant alterations in the quantum yield of oxygen evolution, as evidenced by the light response curves, were observed, indicating that pea plants were able to tolerate 100 mM NaCl for a period of 5 days and that their ability to evolve oxygen was not affected. The presence of 150 mM NaCl nearly eliminated the photosynthetic oxygen evolution after 5 days of treatment, while in the presence of 200 mM NaCl, the damaging effect was detected for both periods of treatment, and no restoration of the oxygen-evolving ability was achieved after removal of NaCl.

### 3.3. Photochemical Activity of PSII and PSI Affected by the Salt Application

The photochemical activities of both photosystems, PSII and PSI, were assessed in the presence of artificial electron donors and acceptors [51]. The presence of NaCl negatively affected the electron transport through the reaction center of PSII in a concentration-dependent manner and to a higher extent after the longer period of treatment. The reduction could be due to either the attack of the reaction center (D1) of PSII [52,53] and/or to the damage of the OEC, situated on the donor side of PSII [3,11]. Removing the salt from the medium led to a complete restoration of PSII activity (100 mM NaCl) to the values of the thylakoids of control plants. After treatment with 150 mM NaCl, no restoration of PSII activity was detected, probably due to residual stress related processes and/or irreversible damage as a result of ionic stress. With respect to activity of PSI, a certain degree of stimulation was detected after 2 days of treatment with 100 and 150 mM NaCl. A similar stimulation of PSI-mediated electron transport was reported for the thylakoids of tomato plants treated with a high temperature and high light intensity [54] and for *Arabidopsis thaliana* subjected to high light at low temperature [38]. The observed stimulation could be due to the activation of a cyclic electron flow around PSI, providing additional ATP for metabolic reactions and maintaining the proton gradient across the thylakoid membrane [55]. It was reported that NaCl did not affect the activity of PSI [32], but our results indicated that the ability of PSI reaction center to transfer electrons was seriously affected and did not recover (treatment with 150 mM NaCl) upon salt removal, similar to the lack of recovery of the photochemical activity of PSII. The NaCl-induced inactivation of the PSII and PSI centers was proposed in the hypothetical model for the effect of salt on cyanobacterial cells [3,11]. Inhibition of the activity of both photosystems was also detected after salt treatment of *Paulownia elongate x elongate* and *Paulownia tomentosa x fortune* [56].

### 3.4. Activity of the Oxygen-Evolving Complex Affected by Salt Stress

The reduction in the photochemical activity of PSII correlated with the obtained oxygen flash yields that are believed to characterize the functioning of OEC of PSII centers situated in the grana regions (PSIIα). The decrease in the amplitudes after 5 days of treatment with increased concentrations of NaCl was more strongly expressed in comparison with the second day of exposure. For all investigated thylakoid membranes isolated from control, treated and recovered plants, the oscillation pattern of the registered oxygen flash yield was preserved with a maximum at the third and seventh flash, indicating that the activity of PSIIα centers that remained active was preserved. In addition, the decrease in the A_1_/A_2_ ratio indicated that the contribution of PSIIα centers to the overall evolved oxygen was gradually decreased with the elevation in NaCl concentration and duration of treatment, suggesting that these centers were stronger affected by the salt treatment in comparison with PSIIβ. Similar higher sensitivity of PSIIα centers was reported for pea plants after heat treatment and fluidity modification [37], as well as after UV treatment [39], and for *Arabidopsis thaliana*, wt and *lut2* mutant, subjected to simultaneous treatment with high light and low temperature [38]. The kinetics of both types of centers were also affected by the salt treatment, expressed as the retardation of their turnover, as evidenced by the increase in the time constants of the “fast” PSIIα and “slow” PSIIβ centers, t_1_ and t_2_, respectively. The observed reduction in the activity of OEC of both populations of PSII is in accordance with reported data, that the extrinsic proteins of the super complex of PSII, the OEC, were dissociated from and/or inactivated by salt stress [3,11]. The results presented here indicate that the presence of 100 mM NaCl slightly affected the estimated parameters after analysis of the decay kinetics of the oxygen burst under continuous illumination (A_1_/A_2_, t_1_ and t_2_), but after recovery, no restoration to the values of thylakoids from the non-treated plants was achieved.

### 3.5. Salt-induced Alterations in Energy Transfer between the Main Pigment–Protein Complexes

The energy transfer within the multiprotein complex of PSII and between PSII and PSI after the salt treatment was assessed by the low (77K)-temperature fluorescence emission spectra of isolated thylakoid membranes. The transfer of energy in the complex of PSII from CP47 to the reaction center, reflected by the ratio F685/F695, was decreased only after prolonged treatment with 150 and 200 mM NaCl, supposing that the energy transfer within the PSII complex was disturbed by the salt treatment. This result is in accordance with the suggestion that one of the consequences of salt stress is the attack of CP47 and its disconnection from the reaction center of PSII, as reviewed in [11]. The fluorescence emission of the PSI complex was slightly increased as a result of plants exposure to 150 and 200 mM NaCl for 2 days and to 150 mM NaCl for 5 days, as evidenced by the slightly elevated ratio of F735/F685. This could be due to either more energy being delivered to PSI or the transfer of energy to PSI complex by the LHCII of PSII. For the highest concentration of NaCl, the ratio F735/F685 was decreased, most probably due to less energy being delivered to PSI or to the destruction of the whole complex. Indeed, salt stress led to the dissociation of plastocyanin and/or cytochrome c553 from PSI, thus inhibiting its activity [11]. Both investigated fluorescent ratios remained unaltered after application of 100 mM NaCl. After the removal of NaCl, the ratios of F685/F695 and F735/F685 of thylakoids from plants previously treated with 150 mM NaCl were not restored to the values of the non-treated plants, suggesting that the integrity of the super complex of PSII and of PSI was not achieved due to increased concentrations of ions in the cells, i.e., ionic stress.

## 4. Materials and Methods

### 4.1. Plant Growth and Treatment

The seeds of pea (*Pisum sativum* L. *cv. Ran 1*) were surface-sterilized with ethanol (96% *v*/*v*) for 5 min, washed several times with water and germinated for 2 days between two wet filter papers. The seedlings were transferred to containers containing 50% Hoagland’s solution and grown for 14 days on a hydroponic culture in a growth chamber (Fytoscope FS130, Photon System Instruments, Drasov, Czech Republic) under controlled conditions: 16 h light/8 h dark, 200 μmol photons m^−2^ s^−1^ at day/night temperatures of 24/18 °C and a humidity of 70%. The plants were treated with different concentrations of NaCl (100, 150, 200 mM), which were added to the Hoagland’s solution. The addition of NaCl was performed in portions until the required concentration was reached. Every second day, the solution, without or with added NaCl, was replaced with a fresh one. Leaves for analysis were taken from the pea control plants, grown without salt during the whole experimental setup, after 2 and 5 days of treatment with NaCl and after 6 days of recovery on pure 50% Hoagland’s solution. Three independent experiments were performed. The complete premature senescence and dehydration of the plant leaves prevented the presentation of results in some cases.

### 4.2. Determination of the Relative Water Content (RWC)

The relative water content (RWC; %) of the pea leaves from the control, treated and recovered plants was determined gravimetrically as:

RWC [%] = 100 × (FM − DM)/(SM − DM)

The fresh mass (FM) of 8 leaf discs (diameter 14 mm) from different plants was determined immediately after cutting. The saturated mass (SM) was determined after floating the discs on dd.H_2_O for 24 h in the dark at 4 °C, and the dry mass (DM) of the same discs was determined after dehydration in an oven at 85 °C until a constant weight was reached [57]. Three independent experiments were performed, with three parallel samples evaluated at each time point (*n* = 9).

### 4.3. Electrolyte Leakage

The extent of electrolyte leakage (EL) from the leaf tissues was determined by transferring pieces of leaves from different plants (150 mg) to 15 mL dd. H_2_O. The samples were gently shaken for 24 h at room temperature, and the conductivity of the floating solution was determined using a conductometer (Hydromat LM302, Witten, Germany). The maximum leakage was registered after boiling the leaf material at 100 °C for 15 min. The extent of EL was presented as the percent of maximum leakage after boiling. Three independent experiments were conducted, with three parallel samples evaluated at each time point (*n* = 9).

### 4.4. Pigment Content of the Whole Leaves

Pieces of leaves from different control, treated and recovered plants (40 mg) were homogenized in 80% acetone, followed by centrifugation at 4500× *g* for 15 min at 4 °C. The pigment content was determined spectrophotometrically (UV-VIS Specord 210 Plus, Analytic Jena, Jena, Germany) in the clear extract using the formulas reported by Lichtenthaler (1987) [58]. The whole procedure was performed at 4 °C and under dim light. Three independent experiments were conducted, with four parallel samples evaluated at each time point (*n* = 12). The results were presented as the amount of piment per g of fresh weight (FW) (mg pigment g^−1^ FW).

### 4.5. Lipid Peroxidation

The extent of salt-induced lipid peroxidation was evaluated by the thiobarbituric acid method [59]. Leaf material from different of control, treated and recovered plants (100 mg) were homogenized in 3 mL of 0.1% (*w*/*v*) trichloracetic acid (TCA) at 4 °C and centrifuged at 4500× *g* for 15 min. A total of 1 mL of the clear supernatant was mixed with the same volume of 20% TCA containing 0.5% thiobarbituric acid (TBA), followed by the boiling of the mixture for 25 min in a water bath. The absorbance of the clear mixture was recorded at 532 and 600 nm after cooling to room temperature. The amount of malondialdehyde (MDA) formed was determined using the extinction coefficient 155 mM^−1^ and expressed on the basis of the fresh weight as [nmol MDA g^−1^ FW]. Three independent experiments were conducted, and four parallel samples were evaluated at each time point (*n* = 12).

### 4.6. Oxygen-Evolving Activity of the Whole Leaves

The net rate of photosynthetic oxygen evolution was determined using a Clark-type oxygen electrode (model DW1, Hansatech Instruments, King’s Lynn, Norfolk, UK) equipped with an LD1/2 leaf-disc electrode chamber [60]. Every sample contained 8 leaf discs, with a total area of 10 cm^2^. The measurements were performed at room temperature (22 °C) in a saturating atmosphere of CO_2_ provided by 200 µL of 1 M NaHCO_3_. Every sample was dark-adapted for 5 min. The oxygen evolution was registered for 5 min under increasing light intensity (0–1400 μmol photons m^−2^ s^−1^) from red-emitting diodes (>650 nm) (Model LH36/2R, Hansatech Instruments Ltd., King’s Lynn, Norfolk, UK). Light response curves were obtained by plotting the evolved oxygen against the increasing light intensities. Three independent experiments were performed, and two parallel samples were evaluated at each time point (*n* = 6).

### 4.7. Isolation of the Thylakoid Membranes

Thylakoid membranes were isolated from the leaves of the control (non-treated) plants and plants treated for 2 and 5 days with different concentrations of NaCl and after 6 days of recovery on pure Hoagland’s solution, according to the procedure described previously [51], at 4 °C and under dim light. The isolated membranes were dissolved in a medium containing 0.33 M sucrose, 10 mM Tricine (pH 7.5), 5 mM MgCl_2_ and 10 mM NaCl. The pigment content of the isolated thylakoid membranes was determined in 80% acetone extract, according to [58], using a spectrophotometer (UV-VIS Specord 210 Plus, Analytic Jena, Jena, Germany).

### 4.8. Photochemical Activities of PSII and PSI in the Isolated Thylakoid Membranes

The photochemical activity of PSII and PSI in the isolated thylakoid membranes of the control, treated and recovered pea plants was determined using a Clark-type oxygen electrode (DW1, Hansatech Instruments Ltd.) under saturating white light (1200 μmol photons m^−2^ s^−1^) provided by an LED source in the presence of artificial electron donors and acceptors, as described in [61]. The determination of the oxygen uptake (PSI) and evolution (PSII) was performed at room temperature (22 °C) in a temperature-controlled cuvette at a chlorophyll concentration of 25 µg Chl mL^−1^. The reaction medium for the determination of the PSI activity contained 0.33 M sucrose, 5 mM MgCl_2_, 10 mM NaCl, 20 mM Tricine (pH 7.5), 0.4 μM DCMU, 0.5 mM NH_4_Cl and 5 mM NaN_3_, with the addition of an electron donor of 0.1 mM DCPIP reduced by 4 mM Na ascorbate and an electron acceptor of 0.1 mM MV. The activity of PSII was determined in a medium containing 0.33 M sucrose, 5 mM MgCl_2_, 10 mM NaCl and 20 mM MES (pH 6.5) in the presence of 0.4 mM 1-4 BQ. Three independent experiments were conducted, with three parallel samples evaluated at each time point (*n* = 9).

### 4.9. Measurement of the Oxygen Flash Yields and Initial Oxygen Burst

The evaluation of the activity of the oxygen-evolving complex was performed through the detection of the oxygen flash yields and initial oxygen burst, without the addition of an electron acceptor, using home-constructed equipment with a Joliot-type oxygen electrode [62], as described in [38]. Each sample contained 100 μL of thylakoid membrane at a concentration of 300 µg Chl mL^−1^. The reaction medium contained 0.33 M sucrose, 5 mM MgCl_2_, 10 mM NaCl and 20 mM MES (pH 6.5). Each sample was pre-illuminated with 25 flashes, followed by a 5 min dark adaptation. The calculation of the oxygen flash yields was performed according to the non-cooperative Kok’s model of oxygen evolution [63]. The kinetic parameters of the initial oxygen burst under continuous illumination, amplitudes and time constants (A_1_, A_2_, t_1_ and t_2_) were evaluated by the fitting of the oxygen burst decay curve with two exponential components. Three independent experiments were performed, with two parallel samples evaluated at each time point (*n* = 6).

### 4.10. 77K Fluorescence Measurements

The energy transfer between the main pigment–protein complexes in the thylakoid membranes from the control, treated and recovered pea plants was determined by recording and analyzing the low-temperature (77K) fluorescence emission spectra using a Jobin Yvon JY3 spectrofluorometer (Division d’Instruments S.A., Longjumeau, France) equipped with a red-sensitive photomultiplier (Hamamatsu R928, Hamamatsu Photonics, Japan) and a low-temperature device. The thylakoid membranes, at a concentration of 10 µg Chl mL^−1^, were resuspended in a medium containing 0.33 M sucrose, 5 mM MgCl_2_, 10 mM NaCl and 20 mM Tricine (pH 7.5). Each sample was transferred to a quartz tube for the fluorescence measurements and immediately frozen in liquid nitrogen [38]. Fluorescence emission spectra were recorded after excitation with 436 nm to preferentially excite Chl a. The width of the slits was 4 nm. The spectra were analyzed using Origin 7.0. Three independent experiments were conducted, with two parallel samples evaluated at each time point (*n* = 6).

### 4.11. Statistics

The data were presented as mean values ± SE. Statistically significant differences between the values of the control (0 mM NaCl) plants and plants treated with increasing concentrations of NaCl for every time point of the experimental setup (2 and 5 days of treatment and 6 days of recovery) were evaluated using two-way ANOVA followed by Tukey’s post hoc test. The assumptions of the normality of the raw data (using the Shapiro–Wilk test) and the homogeneity of the variances (using Levene’s test) were checked before the ANOVA test’s performance. The homogeneity of variance test was applied to verify the parametric distribution of the data. The values were considered statistically different at *p* < 0.05 after Fisher’s least significant difference post hoc test, using Origin 9.0 for data analysis and graphing software, version 9 (OriginLab, Northampton, MA, USA).

## 5. Conclusions

Salts stress is one of the most extreme environmental conditions affecting plants. The exposure of pea plants (*Pisum satimum L*. *cv. Ran 1*) to three different concentrations of NaCl for up to 5 days affected a number of parameters, including the water content, membrane stability, extent of lipid peroxidation and pigment content, as well as the photosynthetic performance, primary photosynthetic reactions and energy transfer between the pigment–protein complexes. The exposure of the pea plants to 100 mM NaCl led to a small decrease in the water content of the leaves and significant disturbance of the membrane integrity, but the lipid peroxidation was not affected. The complete recovery of the RWC and membrane integrity was achieved on the removal of NaCl from the growth medium. In addition, the oxygen-evolving activity of the whole leaves, activity of the PSIIα centers situated in the grana regions of the thylakoid membranes, energy interaction within the multiprotein complex of PSII and energy delivery to PSI were only slightly affected by 100 mM NaCl for 5 days of treatment. We consider that during the 5 days of exposure to 100 mM NaCl, the generated reactive oxygen species were successfully scavenged by the antioxidant system, and the plants were able to recover. The application of higher concentrations of NaCl (150 and 200 mM NaCl) led to a significant concentration-dependent water loss, disturbance of the membrane integrity, lipid peroxidation and increase in the pigment content. The net photosynthetic rate, electron transport through the reaction centers of PSII and PSII, activity of the PSIIα centers and energy transfer between the pigment–protein complexes were also negatively affected. After the recovery period, the above-listed parameters were not restored to the values of the control plants, indicating that either the activity of the antioxidant system of the plants was not able to scavenge the stress-generated ROS and/or the accumulation of salt ions in the cells was too high, leading to ionic stress. The investigated *Pisum sativim L. cv. Ran 1* was tolerant of the presence of 100 mM NaCl for a short period of time (5 days) and was able to recover after the salt stress, while the treatment for the indicated time period with higher concentrations of NaCl was detrimental to the pea plants due to ionic stress.

## Data Availability

Not applicable.
